# Systemic inflammatory response syndrome criteria and the prediction of hospital mortality in critically ill patients: a retrospective cohort study

**DOI:** 10.5935/0103-507X.20170047

**Published:** 2017

**Authors:** Leandro Utino Taniguchi, Ellen Maria Campos Pires, José Mauro Vieira Jr., Luciano Cesar Pontes de Azevedo

**Affiliations:** 1 Research and Education Institute, Hospital Sírio-Libanês - São Paulo (SP), Brazil.; 2 Emergency Medicine Discipline, Faculdade de Medicina, Universidade de São Paulo - São Paulo (SP), Brazil.

**Keywords:** Systemic inflammatory response syndrome, Mortality, Prognosis, Infection, Sepsis

## Abstract

**Objective:**

This study intended to determine whether the systemic inflammatory response syndrome criteria can predict hospital mortality in a Brazilian cohort of critically ill patients.

**Methods:**

We performed a retrospective cohort study at a private tertiary hospital in São Paulo (SP), Brazil. We extracted information from the adult intensive care unit database (Sistema Epimed™). We compared the SAPS 3 and the systemic inflammatory response syndrome model as dichotomous (≥ 2 criteria: systemic inflammatory response syndrome -positive *versus* 0 - 1 criterion: systemic inflammatory response syndrome -negative) and ordinal variables from 0 to 4 (according to the number of systemic inflammatory response syndrome criteria met) in the prediction of hospital mortality at intensive care unit admission. Model discrimination was compared using the area under the receiver operating characteristics (AUROC) curve.

**Results:**

From January to December 2012, we studied 932 patients (60.4% were systemic inflammatory response syndrome -positive). systemic inflammatory response syndrome -positive patients were more critically ill than systemic inflammatory response syndrome -negative patients and had higher hospital mortality (16.9% *versus* 8.1%, p < 0.001). In the adjusted analysis, being systemic inflammatory response syndrome -positive independently increased the risk of death by 82% (odds ratio 1.82; 95% confidence interval [CI] 1.12 - 2.96, p = 0.016). However, the AUROC curve for the SAPS 3 model was higher (0.81, 95%CI 0.78 - 0.85) compared to the systemic inflammatory response syndrome model with the systemic inflammatory response syndrome criteria as a dichotomous variable (0.60, 95%CI 0.55 - 0.65) and as an ordinal variable (0.62, 95%CI 0.57 - 0.68; p < 0.001) for hospital mortality.

**Conclusion:**

Although systemic inflammatory response syndrome is associated with hospital mortality, the systemic inflammatory response syndrome criteria show low accuracy in the prediction of mortality compared with the SAPS 3.

## Abstract (pt)

**Objetivo:**

Determinar se os critérios para definição de síndrome de resposta inflamatória sistêmica podem predizer a mortalidade hospitalar em uma coorte brasileira de pacientes críticos.

**Métodos:**

Conduzimos um estudo retrospectivo de coorte em um hospital terciário privado localizado na cidade de São Paulo (SP). Extraímos as informações da base de dados de uma unidade de terapia intensiva para adultos (Sistema Epimed™). Comparamos o SAPS 3 e o modelo da síndrome de resposta inflamatória sistêmica de forma dicotomizada (≥ 2 critérios, para síndrome de resposta inflamatória sistêmica positiva, em comparação com zero a um critério, para síndrome de resposta inflamatória sistêmica negativa) e variáveis ordinais de zero a 4 (segundo o número de critérios preenchidos para síndrome de resposta inflamatória sistêmica) para predição de mortalidade hospitalar por ocasião da admissão à unidade. A discriminação do modelo foi comparada com uso da área sob a curva *receiver operating characteristics* (ASCROC).

**Resultados:**

Entre janeiro e dezembro de 2012, estudamos 932 pacientes (60,4% deles eram síndrome de resposta inflamatória sistêmica positiva). Os pacientes positivos para síndrome de resposta inflamatória sistêmica estavam em estado crítico mais grave do que os negativos, e tiveram mortalidade hospitalar mais elevada (16,9% *versus* 8,1%; p < 0,001). Na análise ajustada, ser síndrome de resposta inflamatória sistêmica positivo aumentou de forma independente o risco de óbito em 82% (OR 1,82; IC95% 1,12 - 2,96; p = 0,016). Entretanto, a ASCROC para os critérios do modelo SAPS 3 foi mais elevada (0,81; IC95% 0,78 - 0,85) em comparação ao modelo síndrome de resposta inflamatória sistêmica, tendo os critérios para síndrome de resposta inflamatória sistêmica de forma dicotomizada (0,60; IC95% 0,55 - 0,65) e como variável ordinal (0,62; IC95% 0,57 - 0,68; p < 0,001) para mortalidade hospitalar.

**Conclusão:**

Embora a síndrome de resposta inflamatória sistêmica se associe com mortalidade hospitalar, os critérios para esta síndrome tiveram baixa acurácia para predição da mortalidade, quando comparados ao SAPS 3.

## INTRODUCTION

In 1992, an American consensus statement was published. The term systemic inflammatory response syndrome (SIRS) was developed, including a definition of sepsis as the presence of this systemic inflammatory response as a result of infection.^(1)^ Since this time, more than 100 clinical trials have used these criteria for the inclusion of patients,^(2)^ including recently published trials.^(3-5)^ However, the utility of the SIRS criteria for the selection of a more critically ill group of patients who are expected to benefit from early identification and timely intervention remains controversial. In 1995, Rangel-Frausto et al. showed that up to 64% of ward patients have SIRS during their hospital stay.^(6)^ More recently, Churpek et al. demonstrated an incidence of SIRS of nearly 50% in ward patients.^(7)^ These findings support the low specificity of the SIRS criteria for the selection of patients at a higher risk of death because most hospitalized patients develop SIRS at some point during their stay. Finally, Kaukonen et al. concluded that the SIRS criteria missed one in eight patients with severe sepsis, challenging the notion of the high sensitivity of the available criteria for the definition of sepsis at that time.^(8)^

Some authors have advocated the systematic documentation of SIRS status upon hospital admission to guide clinical decisions regarding the presence of infection and prognosis.^(9)^ However, SIRS may occur in association with common non-infectious conditions, such as high-risk surgery^(10)^ and trauma.^(11)^ In fact, mortality rates are similar between infectious and non-infectious conditions associated with SIRS.^(12)^ Therefore, the SIRS criteria alone may not effectively discriminate between infected and non-infected patients.

This study intended to determine whether the systemic inflammatory response syndrome criteria can predict hospital mortality in a Brazilian cohort of critically ill patients.

## METHODS

We performed a retrospective cohort analysis of patients who were admitted to the 30-bed, mixed, medical-surgical intensive care unit (ICU) of *Hospital Sírio-Libanês*. This hospital is a private tertiary hospital with a dedicated cancer center in São Paulo, Brazil. Cardiac surgical patients are managed in a separate unit of our hospital. The hospital has a step-down unit with 31 beds, 24-h availability of an intensivist, and a higher nurse-patient ratio than the ward at this step-down unit. The study was approved by the local institutional Ethics Committee (number CAAE: 42763115.7.0000.5461), which waived informed consent due to the observational design of the study.

Our analysis used anonymized administrative data that were prospectively collected at ICU admission in a software database (Sistema Epimed™; www.epimedmonitor.com). The study population consisted of all consecutive adult patients (older than 18 years) who were admitted between January 1^st^, 2012 and December 31, 2012 and they all had variables for the SIRS criteria that were collected at ICU admission. The exclusion criteria were an ICU length of stay (LOS) shorter than 24 hours (to exclude patients admitted for minor procedures, such as cardiac catheterization), pregnancy, and refusal of invasive procedures because of palliative care. Patients who were transferred from other hospitals were excluded. If patients had more than one admission during the inclusion period, only the first admission was included.

Systemic inflammatory response syndrome was defined as fulfilling at least two of the following four criteria: (1) fever > 38.0°C or hypothermia < 36.0°C; (2) tachycardia > 90 beats/minute; (3) tachypnea > 20 breaths/minute; and (4) leukocytosis > 12×10^9^/L or leucopenia < 4×10^9^/L.^(1)^ Vital signs were collected by registered nurses at ICU admission. Blood samples for leucocyte counts were collected within the first hours of admission. Documentation of the presence of suspected or confirmed infection was based on a clinical evaluation within the first day of ICU admission, including clinical examinations and radiological evaluations, and when infection was suspected by the intensive care physician or was indicated by blood, urine, and other cultures.^(9)^ To check the concordance between infection information in the database and patient charts, one of the authors randomly audited 300 charts blinded to the SIRS criteria. Concordance between the database and the patient chart for the presence of suspected infection at ICU admission was observed in 290 cases (96.6%). All patients with suspected infection received antibiotics on the first day of admission.

The recorded data included age, sex, the Simplified Acute Physiology Score 3 (SAPS 3),^(13,14)^ the referring unit, diagnosis at admission, surgical procedures before admission, the presence and types of comorbidities, the length of hospital stay before ICU admission, the presence and type of SIRS criteria, and the resources used at ICU admission (invasive mechanical ventilation, vasoactive drugs, or renal replacement therapy). The follow-ups for the patients in our database were determined relative to the duration of ICU and hospital stays and hospital mortality.

### Statistical analysis

Normality of distribution was verified with the Kolmogorov-Smirnov test for continuous variables. The data are presented as the mean (SD) and the median (25^th^ percentile - 75^th^ percentile) for parametric and nonparametric variables, respectively. Categorical variables are presented as rates or percentages. A comparison of the parametric variables between the groups was performed with the unpaired Student's *t*-test and a comparison within the groups was performed with the paired Student's *t*-test. Non-parametric variables were compared using the Mann-Whitney test. All statistics were two-tailed and a p value < 0.05 was considered statistically significant.

Patients were categorized at ICU admission as SIRS-positive or SIRS-negative if they presented with two or more SIRS criteria or with one or none of the criteria, respectively. To identify independent differences at ICU admission between patients who were SIRS-positive and SIRS-negative, we performed a multivariable logistic regression with SIRS-positive as the outcome. Variables with p values < 0.1 in the univariate analysis were included in the logistic model. The model was refined using the backward stepwise likelihood ratio method, excluding the least significant variable at each step. Using this prediction model, we estimated the probability of being SIRS-positive for each patient.^(8)^ This information was generated to take imbalances between SIRS-positive and SIRS-negative patients into account as previously performed.^(8)^

To evaluate the independent predictive capacity of the SIRS criteria to identify a higher risk of death, we performed a multivariate logistic regression analysis with hospital mortality as the dependent factor. Systemic inflammatory response syndrome was considered in the model as a categorical dichotomous variable (SIRS-positive *versus* SIRS-negative) and as an ordinal variable (0 - 4, reflecting the number of SIRS criteria met). In the model, we adjusted for the severity of illness using a modified risk of death estimation ("modified SAPS 3", i.e., the original SAPS 3 model without body temperature, heart rate, or leukocytes because they are also SIRS criteria) in conjunction with the probability of being SIRS-positive to adjust for baseline differences.^(8)^ The model was also refined using the backward stepwise likelihood ratio method, excluding the least significant variable at each step. All included variables had less than 3% missing data, and no imputation was performed for missing values. The discrimination of the model for hospital mortality was evaluated with the area under the receiver operating characteristics (ROC) curve (AUC). A comparison was performed between SIRS criteria models as categorical and ordinal variables without adjustment and the SAPS 3. A comparison between the AUC values was performed as described by DeLong et al.^(15)^ Finally, we evaluated the predictive capacity of the SIRS criteria for the presence of suspected or confirmed infection at ICU admission using sensitivity, specificity, predictive values and likelihood ratios (LR) of the SIRS criteria. The data were analyzed using IBM Statistical Package for Social Science (SPSS) for Windows, Version 20.0 (IBM Corp., Armonk, NY, USA) and MedCalc Statistical Software version 16.8 (MedCalc Software, Ostend, Belgium).

## RESULTS

**Figure 1:**
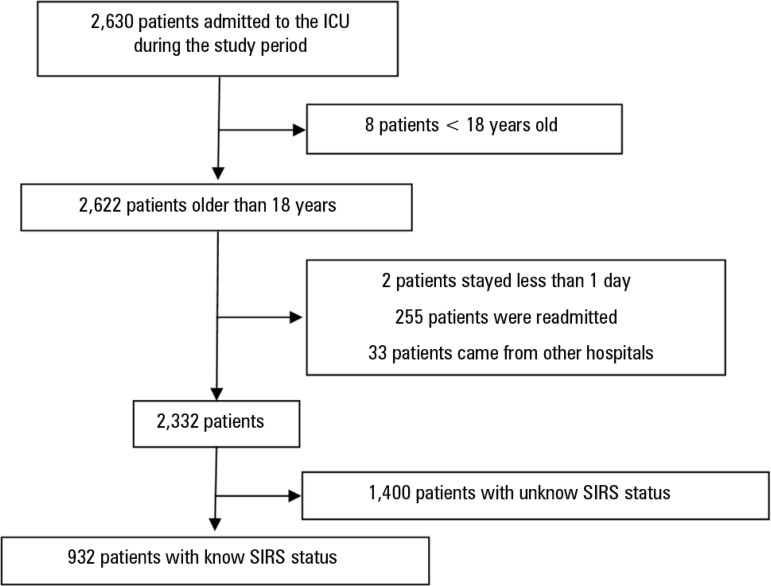
Patient flow diagram of the study. ICU - intensive care unit; SIRS - systemic inflammatory response syndrome.

During the study period, 2,332 patients were admitted and did not have any exclusion criteria (Figure 1). At ICU admission, SIRS status could be evaluated in 932 (40%) patients. Systemic inflammatory response syndrome status was unknown in 1,400 patients, largely because of a lack of data on leukocytes in our database (Electronic Supplementary Material - Table S1). There was a small, but significantly different, difference of SAPS 3 values, but not hospital mortality, between patients with known and unknown SIRS status (Electronic Supplementary Material - Table S1). The results presented here are based on patients with known SIRS status at ICU admission.

The general characteristics of the patients are shown in table 1. Of the patients with known SIRS status at ICU admission, 563 (60.4%) were SIRS-positive and 369 (39.6%) were SIRS-negative. Systemic inflammatory response syndrome-positive patients were more frequently male with a higher severity of illness (higher SAPS 3 and more invasive procedures required, such as invasive mechanical ventilation and vasoactive drugs) compared to SIRS-negative patients. Systemic inflammatory response syndrome-positive patients also more frequently came from the wards because of medical causes after a longer hospital stay before ICU admission than SIRS-negative patients. Infection was more prevalent at ICU admission and hospital mortality was higher in the SIRS-positive patients compared to the SIRS-negative patients. Independent risk factors for being SIRS-positive are shown in Electronic Supplementary Material - Table S2.

**Table 1:** The general characteristics of patients at intensive care unit admission and hospital mortality rates

	All patients	SIRS-positive	SIRS-negative	p value*
N	932	563 (60.4)	369 (39.6)	
Age (SD) (years)	66.2 (17.8)	65.6 (17.7)	67.1 (18)	0.19
Male	520 (55.8)	309 (54.9)	211 (57.2)	0.001
SAPS 3	42 [33 - 53]	45 [34 - 56]	39 [31 - 50]	< 0.001
Admission type				< 0.001
Medical	453 (48.6)	306 (54.2)	159 (43.1)	
Emergency surgery	96 (10.3)	57 (10.2)	33 (9.0)	
Elective surgery	383 (41.1)	200 (35.6)	177 (47.9)	
Admission source†				< 0.001
Ward	109 (11.7)	79 (14)	30 (8.1)	
Emergency room	186 (20)	113 (20.1)	73 (19.8)	
Operating room	442 (47.4)	242 (43)	200 (54.2)	
Intermediate care	66 (7.1)	47 (8.4)	23 (6.3)	
Length of hospital stay before ICU admission (days)	1 [0 - 2]	1 [0 - 3]	1 [0 - 2]	0.012
Non-oncohematological comorbidities				0.50
0	730 (78.3)	441 (78.4)	300 (81.3)	
1	160 (17.2)	99 (17.5)	55 (14.9)	
≥ 2	42 (4.5)	23 (4.1)	14 (3.8)	
Cancer	466 (50)	289 (51.3)	177 (48)	0.10
Infection at admission‡	183 (19.6)	132 (23.4)	50 (13.5)	< 0.001
Mechanical ventilation	198 (21.2)	138 (24.5)	60 (16.3)	0.001
Vasoactive drugs	326 (35)	221 (39.3)	105 (28.5)	< 0.001
Dialysis	57 (6.1)	39 (6.9)	18 (4.9)	0.15
Hospital mortality	125 (13.4)	95 (16.9)	30 (8.1)	< 0.001

SIRS - systemic inflammatory response syndrome; SD - standard deviation; SAPS 3 - Simplified Acute Physiology Score 3.

* p value for the comparison between systemic inflammatory response syndrome-positive and systemic inflammatory response syndrome-negative groups;

† 13.8% of the patients were from other areas of the same hospital (e.g., interventional radiology room);

‡ infection at admission was confirmed or suspected by attending physicians. Systemic inflammatory response syndrome-positive was defined as patients with two or more criteria for systemic inflammatory response syndrome. SIRS-negative was defined as patients with one or no criteria. Results are expressed as N (%) or the median [25^th^ - 75^th^].

The distribution of the SIRS criteria are shown in table 2. The most frequent positive criterion among SIRS-positive patients was respiratory rate, followed by leukocyte count and heart rate. In SIRS-negative patients, the most commonly found single criterion was heart rate, followed by leukocyte count and temperature. The median values of the observed criteria are shown in table 2.

**Table 2:** Distribution of the systemic inflammatory response syndrome criteria among the patients according to positive or negative status

	All patients	SIRS- positive	SIRS-negative	p value*
SIRS criteria				
Increased heart rate	368 (39.5)	323 (57.4)	45 (12.2)	< 0.001
Increased respiratory rate	499 (53.5)	397 (70.5)	102 (27.6)	< 0.001
Abnormal temperature	312 (33.5)	255 (45.3)	57 (15.4)	< 0.001
Abnormal leukocyte counts	439 (47.1)	373 (66.3)	66 (17.9)	< 0.001
Number of SIRS criteria	2 [1 - 2]	2 [2 - 3]	1 [0 - 1]	< 0.001
Zero	99 (10.6)	-	99 (26.8)	
One	270 (29)	-	270 (73.2)	
Two	375 (40.2)	375 (66.6)	-	
Three	154 (16.5)	154 (27.4)	-	
Four	34 (3.6)	34 (6)	-	
SIRS criteria				
Heart rate (beats/minute)	84 [73 - 98]	94 [79.8 - 107]	77 [68 - 85]	< 0.001
Respiratory rate (breaths/minute)	20 [16 - 24]	22 [19 - 26]	17 [15 - 20]	< 0.001
Temperature (ºC)	36.2 [35.8 - 36.6]	36 [35.6 - 36.6]	36.3 [36 - 36.6]	< 0.001
Leukocytes × 10^3^/mm^3^	10.4 [7.4 - 14.1]	12.5 [8.14 - 16.3]	8.8 [7.0 - 11.0]	< 0.001

SIRS - systemic inflammatory response syndrome.

* p value for the comparison between systemic inflammatory response syndrome -positive and negative groups. Patients could have more than one criterion. Results are expressed as N (%) or median [25^th^ - 75^th^ percentiles].

**Figure 2:**
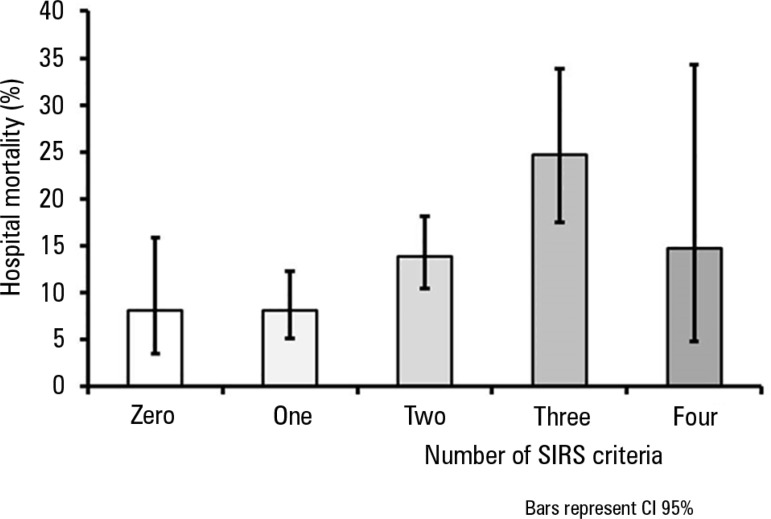
Mortality according to the number of systemic inflammatory response syndrome criteria that were met. SIRS - systemic inflammatory response syndrome; CI95% - confidence interval 95%.

**Figure 3:**
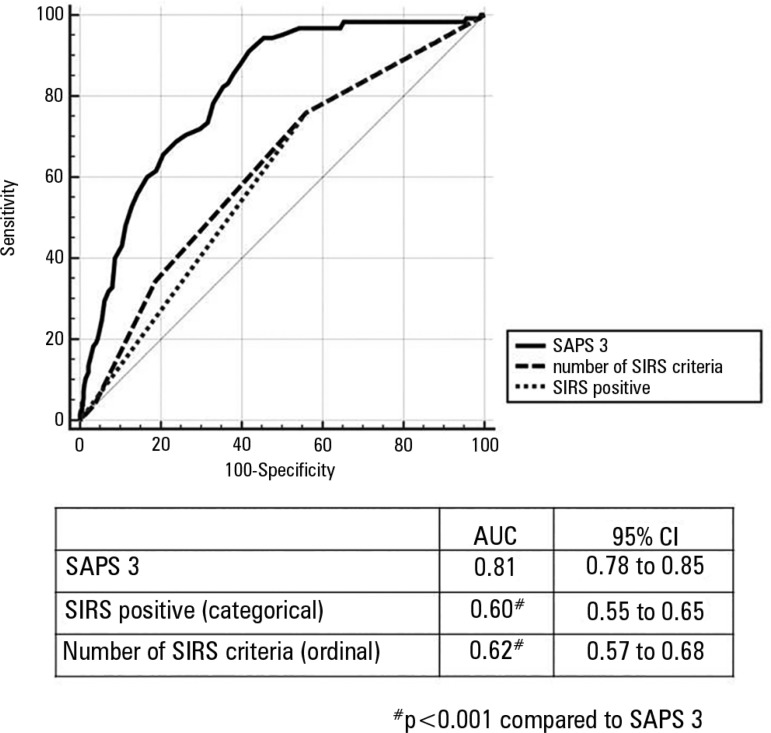
Receiver operating characteristic curves for the prediction of hospital mortality. AUC - area under the curve; CI95% - confidence interval 95%; SAPS 3 - Simplified Acute Physiology Score 3; SIRS - systemic inflammatory response syndrome.

The crude hospital mortality rate was higher (16.9%) in SIRS-positive patients compared with SIRS-negative patients (8.1%, p < 0.001). As the number of SIRS criteria that were met increased, crude hospital mortality increased, except between the groups with three and four criteria (probably because of the low number of patients with four SIRS criteria, Figure 2). In the multivariate logistic regression with hospital mortality as the dependent variable, which included the probability of being SIRS-positive (adjusted for baseline differences), the "modified SAPS 3" (SAPS 3 without SIRS criteria), and SIRS status (positive or negative), being SIRS-positive was an independent risk factor for mortality (odds ratio 1.82; 95% confidence interval [CI] 1.12 - 2.96, p = 0.016). In a similar model with SIRS as an ordinal variable (0 - 4 according to the number of criteria met), an increase of 29% in hospital mortality was observed for each criterion (odds ratio 1.29; 95%CI 1.03 - 1.60, p = 0.024) (Electronic Supplementary Material - Table S3). However, the discrimination of hospital mortality was greater for the SAPS 3 model (AUC 0.81, 95%CI 0.78 - 0.85) than for SIRS criteria models as a dichotomous variable (AUC 0.60, 95%CI 0.55 - 0.65) and as an ordinal variable (AUC 0.62, 95%CI 0.57 - 0.68; p < 0.001 for the comparison between the SAPS 3 and SIRS criteria models, Figure 3). The commonly used cutoff for the two criteria of SIRS had a sensitivity of 76% (95%CI 67.5 - 83.2) and a specificity of 44% (95%CI 40.3 - 47.7) to predict hospital mortality in our cohort.

The comparison between the SIRS criteria and the presence of suspected infection at admission is shown in table 3.

**Table 3:** Systemic inflammatory response syndrome criteria and clinical suspicion of infection

SIRS	Suspected infection	No suspicion of infection
Positive	133	420
Negative	56	323

SIRS - systemic inflammatory response syndrome. Sensitivity: 70%; specificity: 43%; positive predictive value: 24%; negative predictive value: 82%; positive likelihood ratio: 1.24, negative likelihood ratio: 0.68.

## DISCUSSION

In our retrospective cohort study, we observed that SIRS was present in the majority of ICU patients and was associated with twice the crude mortality of patients without SIRS. Although the SIRS criteria were independently associated with hospital mortality, discrimination was poor, and it was significantly lower than that with the SAPS 3 score. The typical cutoff for the two SIRS criteria showed a sensitivity of 76% to identify patients with higher mortality, with even worse specificity (< 50%). Finally, the SIRS criteria performed poorly in the identification of patients with suspected infection.

Our results are consistent with previous publications that showed a high prevalence of SIRS status in hospitalized patients, particularly in the ICU.^(6,7,9,12)^ In a seminal study published in 1995, Rangel-Frausto et al. observed incidence density rates in surgical and medical ICUs of 857 and 804 episodes per 1000 patient-days (i.e., patients were SIRS-positive during >80% of their unit stay).^(6)^ More recently, Dulhunty et al. studied 23 Australian and New Zealand ICUs and observed that 88.4% of admitted patients were SIRS-positive and that the SIRS criteria were met on 88.2% of observed days.^(12)^ Because SIRS is highly prevalent in critically ill patients, specificity is expected to be poor.^(16)^ In fact, in our study, the specificity for hospital mortality was only 44%.

Our results suggest an independent association of SIRS as a dichotomous variable and as an ordinal variable with mortality after controlling for other baseline differences. This finding has been previously demonstrated by some^(8,9)^ but not all studies.^(6,17,18)^ Different settings (emergency department, hospital wards, and ICU) and case-mixes (the inclusion of all admitted patients or only infected ones, different countries, and the year of data acquisition) could explain these inconsistencies between studies. However, more importantly, the discrimination (i.e., the ability of the criteria to correctly classify those with and without the condition) of SIRS status was poor in our study. A comparison with SAPS 3 in our study might not be adequate because this prognostic model was specifically developed for the prediction of hospital mortality. However, even without considering that the SAPS 3 outperformed the SIRS criteria in the prediction of mortality, the AUC for SIRS showed confidence intervals as low as 0.55. Therefore, although the SIRS criteria are associated with worse outcomes, these criteria cannot accurately differentiate which patients would have a higher risk of death. This could lead to inappropriate triage and treatment decisions. Indeed, Alberti et al. suggested that higher cutoff values for some of the SIRS components (e.g., heart rate > 120 beats/minute and temperature > 38.2ºC) are needed with variables of organ dysfunction to model a better risk probability for the progression from sepsis to severe sepsis.^(19)^

Finally, our results do not encourage the widespread use of the SIRS criteria alone to identify infection episodes. We observed low specificity in the SIRS criteria. Similarly, our findings also suggest low clinical usefulness, with sensitivities approximately 70%. Although a high (85%) negative predictive value was observed, this could be due to the lower proportion (20%) of patients presenting with suspected or confirmed infection. The likelihood ratio (a measurement of a diagnostic test that is not affected by the prevalence) is close to one. Comstedt et al. found only a moderate association between SIRS status and infection in a medical emergency ward, but discrimination was not described.^(9)^ Another study showed that the presence of two or more SIRS criteria adds little value in the diagnosis of infection, with a sensitivity of 69%, a specificity of 35%, and a positive likelihood ratio near unit.^(20)^ Although the Sepsis-3 Task Force suggested that SIRS is still useful for the identification of infection,^(21)^ the correct identification of patients with infection should probably include inflammatory criteria and other biomarkers.

Our results have some limitations. First, this was a single-center retrospective cohort from a private hospital, which could have biased some of our results and limited generalizability. Our population has a high proportion of elective surgical patients, with a low prevalence of invasive procedures and low illness severity. Caution is advised before the application of our results in different case-mix ICUs. Second, a large proportion of eligible patients were excluded from the analysis because of missing data related to the SIRS criteria. However, patients with known and unknown SIRS status showed similar characteristics at baseline, but most were not included because of a lack of leukocyte counts at ICU admission. Nevertheless, most of them had one leukocyte count within the first 24 hours, but the results are not available in our database. Third, we evaluated patients at ICU admission. Therefore, we cannot make assumptions about events after this period, but they are likely associated with outcomes. Finally, the accuracy of infection status was not independently audited in all charts and some of the infections may not have been confirmed later during admission. However, at the bedside, the diagnosis of infection is usually confirmed retrospectively, and attending physicians need to make clinical decisions based on available data.

## CONCLUSION

The utility of the systemic inflammatory response syndrome criteria in the recognition of the severity of illness and in the prediction of hospital mortality may be limited.
