# Supplementary material for: Systemic inflammatory response syndrome criteria and the prediction of hospital mortality in critically ill patients: a retrospective cohort study
Source: Rev Bras Ter Intensiva. 2017 Jul-Sep;29(3):317–24. doi: 10.5935/0103-507X.20170047 (PMC5632974; doi:10.5935/0103-507X.20170047)
Supplement: Supplementary file 1 [file rbti-29-03-0317-suppl01.pdf]

Systemic inflammatory response syndrome criteria and the prediction of hospital mortality in critically ill patients: a retrospective cohort study

Critérios para síndrome de resposta inflamatória sistêmica e predição de mortalidade hospitalar em pacientes críticos: estudo retrospectivo de coorte

Leandro Utino Taniguchi, Ellen Maria Campos Pires, José Mauro Vieira Jr., Luciano Cesar Pontes de Azevedo

Table S1 - General characteristics of the patients with known and unknown systemic inflammatory response syndrome status at intensive care unit admission

	Known SIRS status	Unknown SIRS status	p value
N	932 (40)	1,400 (60)	
Age (SD) (years)	66.2 (17.8)	67.2 (18)	0.20
Male	55.8	55.8	0.72
SAPS 3	42 [33 - 53]	42 [32 - 50]	0.01
Admission type			0.1
Medical	48.6	49.6	
Urgent surgery	9.9	12.2	
Elective surgery	39.1	38.2	
Length of hospital stay before ICU admission (days)	1 [0 - 2]	1 [0 - 3]	0.26
Mechanical ventilation	21.2	19.6	0.26
Vasoactive drugs	35	31.7	0.10
Dialysis	6.1	5.6	0.49
Hospital mortality	13.4	12.3	0.82

SIRS - systemic inflammatory response syndrome; SD - standard deviation; SAPS 3 - Simplified Acute Physiology Score 3; ICU - intensive care unit. Among the 2,332 patients evaluated, 85.3% had data related to heart rate, respiratory rate and body temperature at ICU admission. Forty percent had leukocytes counts at ICU admission. Numeric values are expressed as N (%), % and the median [25th - 75th percentiles].

Table S2 - Risk factors associated with being systemic inflammatory response syndrome-positive at intensive care unit admission

Parameter	OR	95%CI	p value
Infection at admission	1.51	1.028 - 2.27	0.036
Vasoactive drugs	1.51	1.13 - 2.02	0.006
Admission from the wards	1.86	1.15 - 2.99	0.012
Admission from interventional radiology	0.302	0.096 - 0.96	0.042
Admission from intermediate care unit	9.75	1.26 - 75.31	0.029

OR - odds ratio; 95%CI - 95% confidence interval. Hosmer-Lemeshow χ^2 p = 0.76.

Table S3 - Multivariate models for hospital mortality prediction

Model 1 - Multivariate model with SIRS criteria as a binomial variable (< 2 or ≥ 2 criteria)

Parameter	OR	95%CI	p value
Probability of being SIRS-positive	1.04	1.02 - 1.07	0.001
Modified SAPS 3*	1.07	1.05 - 1.09	< 0.001
SIRS (dichotomous)	1.82	1.12 - 2.96	0.016

OR - odds ratio; 95%CI - 95% confidence interval; SIRS - systemic inflammatory response syndrome; SAPS 3 - Simplified Acute Physiology Score 3. * Modified SAPS 3: SAPS 3 with the exclusion of SIRS components. Hosmer-Lemeshow χ^2 p = 0.015.

Model 2 - Multivariate model with SIRS criteria as an ordinal variable (0 - 4 criteria)

Parameter	OR	95%CI	p value
Probability of being SIRS-positive	1.04	1.02 - 1.07	0.001
Modified SAPS 3*	1.07	1.05 - 1.09	< 0.001
SIRS (ordinal)	1.29	1.03 - 1.60	0.024

OR - odds ratio; 95%CI - 95% confidence interval; SIRS - systemic inflammatory response syndrome; SAPS 3 - Simplified Acute Physiology Score 3. * Modified SAPS 3: SAPS 3 with the exclusion of SIRS components. Hosmer-Lemeshow χ^2 p = 0.15.
